# The Combination of Functional Metagenomics and an Oil-Fed Enrichment Strategy Revealed the Phylogenetic Diversity of Lipolytic Bacteria Overlooked by the Cultivation-Based Method

**DOI:** 10.1264/jsme2.ME14002

**Published:** 2014-05-23

**Authors:** Takashi Narihiro, Aya Suzuki, Kazuaki Yoshimune, Tomoyuki Hori, Tamotsu Hoshino, Isao Yumoto, Atsushi Yokota, Nobutada Kimura, Yoichi Kamagata

**Affiliations:** 1 Bioproduction Research Institute, National Institute of Advanced Industrial Science and Technology (AIST), 2–17–2–1 Tsukisamu-Higashi, Toyohiraku, Sapporo, Hokkaido 062–8517, Japan; 2 Bioproduction Research Institute, National Institute of Advanced Industrial Science and Technology (AIST), Higashi 1–1–1, Tsukuba, Ibaraki 305–8566, Japan; 3 Division of Applied Bioscience, Graduate school of Agriculture, Hokkaido University, Kita 9 Nishi 9, Kita-ku, Sapporo, Hokkaido 060–8589, Japan; 4 Division of Applied Bioscience, Research Faculty of Agriculture, Hokkaido University, Kita 9 Nishi 9, Kita-ku, Sapporo, Hokkaido, 060–8589, Japan

**Keywords:** functional metagenomics, cultivation, lipolytic enzymes, uncultured microbes

## Abstract

Metagenomic screening and conventional cultivation have been used to exploit microbial lipolytic enzymes in nature. We used an indigenous forest soil (NS) and oil-fed enriched soil (OS) as microbial and genetic resources. Thirty-four strains (17 each) of lipolytic bacteria were isolated from the NS and OS microcosms. These isolates were classified into the (sub)phyla *Betaproteobacteria*, *Gammaproteobacteria*, *Firmicutes*, and *Actinobacteria*, all of which are known to be the main microbial resources of commercially available lipolytic enzymes. Seven and 39 lipolytic enzymes were successfully retrieved from the metagenomic libraries of the NS and OS microcosms, respectively. The screening efficiency (a ratio of positive lipolytic clones to the total number of environmental clones) was markedly higher in the OS microcosm than in the NS microcosm. Moreover, metagenomic clones encoding the lipolytic enzymes associated with *Alphaproteobacteria*, *Deltaproteobacteria*, *Acidobacteria*, *Armatimonadetes*, and *Planctomycetes* and hitherto-uncultivated microbes were recovered from these libraries. The results of the present study indicate that functional metagenomics can be effectively used to capture as yet undiscovered lipolytic enzymes that have eluded the cultivation-based method, and these combined approaches may be able to provide an overview of lipolytic organisms potentially present in nature.

Lipolytic enzymes, including esterases (EC 3.1.1.1) and lipases (EC 3.1.1.3), are used in food, chemical, and pharmaceutical industries as well as waste treatment and biofuel production (19, 20, 22, 24). Commercially available bacterial lipolytic enzymes are mainly derived from culturable strains within the phyla *Proteobacteria*, *Firmicutes*, and *Actinobacteria*. For example, lipolytic enzymes purified from the genera *Burkholderia* and *Pseudomonas* are widely used as detergent additives and in organic synthesis (19, 22, 25), and members of the genera *Bacillus*, *Chromobacterium*, *Staphylococcus*, and *Streptomyces* are listed as popular lipolytic enzyme-producing bacteria (19, 20, 24). The cultivation-based screening method has been employed as one of the main strategies to obtain such microbial resources of not only lipolytic enzymes, but also other biocatalysts from microbial ecosystems (42, 52). However, significant proportions of microorganisms in various environment samples (*e.g.*, soil, sediment, sea water, fresh water, and sludge) have yet to be cultivated and remain functionally unknown (41, 44), which implies that there is room for the expansion of microbial resources to obtain better lipolytic enzymes produced by uncultivated microorganisms.

Functional metagenomics has recently been used to expand the availability of biocatalytic enzymes (17, 31, 56). Since this approach allows the step of cultivation to be skipped, the genetic resources of uncultivated microorganisms in complex microbial communities may be directly accessed. To date, many studies have reported the screening of novel lipolytic enzymes from the functional metagenomic libraries of soil (6, 8, 12, 15, 16, 21, 27, 28, 30, 35, 36, 38, 46, 48, 53, 60, 64), marine sediment (23, 26), tidal flat sediments (34), deep-sea water (18), surface seawater (9), drinking water biofilms (12), pond water (45), hot springs (47), and activated sludge (37). However, no studies have yet compared the phylogenetic diversity of genes encoding lipolytic enzymes obtained by functional metagenomics to that of culturable lipolytic strains isolated using traditional cultivation-based screening techniques.

In addition, although the functional metagenomic approach is a powerful screening tool for novel biocatalysts, a large number of metagenomic clones are generally needed to obtain gene fragments encoding target enzymes. To the best of our knowledge, the maximum screening efficiency of lipolytic enzymes (*i.e.*, the ratio of positive lipolytic clones to total metagenomic clones) was shown to be 0.35% in a soil metagenomic library containing 34,560 fosmid clones (15); however, most attempts have reported markedly lower efficiencies (<0.1%) (6, 21, 27, 28, 30, 35, 36, 48, 53, 59). Therefore, a specific biocatalyst-targeted enrichment strategy was applied to improve this efficiency rate (8, 12, 13, 18, 33, 59). Olive oil (8), mineral oil (12), and crude oil (18) have been used as substrates for the metagenomic screening of lipolytic enzymes. In these studies, two phylogenetically novel lipolytic genes were successfully obtained from metagenomic libraries derived from olive oil-enriched soil and water samples (8). However, no comparative study has examined the frequency of positive clones and phylogenetic diversity of the screened lipolytic enzymes between enriched and non-enriched environmental samples.

In this study, we used comprehensive approaches including both functional metagenomics and a cultivation-dependent method combined with oil-fed enrichment to uncover the entire microbial resources of lipolytic enzymes. Forest soil microbial communities serve important ecological functions, including carbon, nitrogen, and phosphorous cycles, plant diversity, and soil fertility (10, 58). Natural (unmanaged) forest soils are considered to contain an arsenal of microbial genetic resources including lipolytic enzymes (27, 38). We here used natural forest soil as a representative community and employed four different procedures: (i) functional metagenomic screening from natural forest soil; (ii) cultivation-based screening from natural forest soil; (iii) functional metagenomic screening from oil-enriched soil; (iv) cultivation-based screening from oil-enriched soil.

## Materials and Methods

### Strains, plasmids, and growth conditions

*E. coli* strain EPI300 (Epicentre, Madison, WI, USA) and the Copy Control pCC1FOS vector (Epicentre) were used to construct metagenomic libraries. The growth conditions of *E. coli* strains were described previously (32).

### Soil sample and enrichment

Soil samples were collected in May 2007 from a forest located in Sapporo, Hokkaido, Japan (43°04′ N, 141°30′ E). The vegetation type was a cool temperate deciduous broadleaf forest (*Quercus crispula* Blume, *Trillium smallii*, *Anemone flaccida*, *Sasa* spp., and ferns of the *Dryopteridaceae* were observed). Pebbles and plant roots were removed from the samples. The moisture content and pH of the soil samples were measured using a previously described method (40). Non-enriched soil samples (hereafter “NS”) were immediately subjected to cell counting, DNA extraction, and lipolytic activity measurements. To prepare the oil-enriched soil sample (hereafter “OS”), 500 g (wet wt) of soil was placed into a 2-L conical glass flask with a silicon cap, and incubated at 30°C for 2 months without continuous aeration and shaking. A total of 2.5 mL of olive oil (Wako Pure Chemical Industries, Osaka, Japan) was added to the soil and mixing by a spoon every second week. The moisture content and pH were maintained at the initial states during the enrichment by adding distilled water and sodium bicarbonate, respectively.

### Lipolytic activity of soil samples

The lipolytic activities of NS and OS were measured by copper soap colorimetry with small modifications (49). Briefly, 1 g (wet wt) of soil was mixed with 1.25 mL of 2% polyvinyl alcohol:olive oil (3:1, v/v) emulsified solution and 1.25 mL of 200 mM potassium phosphate buffer (pH 6.5). The reaction proceeded at 37°C for 20 min and was stopped by the addition of 5 mL of 200 mM potassium phosphate buffer and 5 mL of acetone. After 2.5 mL of isooctane was added, the reaction mixture was vortexed and boiled for 1 min. The upper isooctane phase containing free fatty acids was collected and mixed with 1 mL copper reagent (49). The resultant mixture was vortexed for 1.5 min and the absorbance of the isooctane phase was measured at 715 nm. One unit of lipase activity was defined as 1 μM free fatty acid released for 1 min. Oleic acid was used as the standard of free fatty acids.

### Cell counting and isolation of major lipolytic bacteria

In cell counting and the isolation of lipolytic bacteria, 1 g (wet wt) of a soil sample was sonicated and diluted serially with phosphate-buffered saline (PBS), as described previously (39). Direct total counts of bacteria were measured by the epifluorescence microscopic method described by Yoshida *et al.* (63). Plate counts of culturable lipolytic bacteria were performed using tributyrin agar plates: per L, 5 g (NH_4_)_2_SO_4_; 1 g KH_2_PO_4_; 1 g K_2_HPO_4_; 0.1 g MgSO_4_ 7H_2_O; 5 g tributyrin; 1 g arabic gum (pH 7.0 by NaOH). We initially attempted to prepare the olive oil-containing agar plate for the isolation of lipolytic bacteria and selection of the *E. coli* transformant; however, it was difficult to prepare a homogeneously diffused olive oil agar plate. Due to this obstacle, a tributyrin-containing agar plate was used for the isolation of lipolytic bacteria from NS and OS samples (and also for the selection of the *E. coli* transformant as described below). The inoculated plates were incubated at 30°C for 2 months before counting colony-forming units (CFU). In order to isolate the main lipolytic bacteria in NS and OS communities, single halo-forming colonies were picked up randomly from the “countable plates,” which were inoculated with 100 μL of 10^−3^–10^−4^ diluted samples and contained 3–10 colonies with clear single halos, and subjected to a standard purification procedure with the repeated streaking of tributyrin agar plates. Phylogenetic analysis of the isolates was performed by partial (*ca.* 500 bp) 16S rRNA gene sequencing according to a previously described method (40).

### Metagenomic screening of the lipolytic fosmid clone

DNA extraction, purification, manipulation, and metagenomic library construction were performed using the methods described by Kimura *et al.* (32). To select lipolytic clones from the constructed fosmid libraries, metagenomic clones in *E. coli* EPI300 were cultured on LB agar supplemented with 12.5 μg mL^−1^ chloramphenicol for 3 d at 37°C. The number of clones per plate was adjusted to approximately 1,000 by the dilution of library stocks, and the plates were incubated overnight at 37°C and stored as a “master plate”. The colonies growing on the master plates were replicated on a LB agar plate supplemented with 0.5% tributyrin, 0.1% arabic gum, and chloramphenicol (7). The replicated plates were incubated for 3 d at 37°C, and the metagenomic clones that produced single halos were isolated. The average insert sizes of cloned lipolytic DNA were estimated by *Bam*H1 digested fragment analyses of 16 randomly selected clones (32).

### *In vitro* mutagenesis and sequencing of metagenomic clones

*In vitro* mutagenesis of the selected lipolytic clones was performed by using the EZ::TN KAN-2 transposon kit (Epicentre) according to a previously described method (32). Briefly, positive *E. coli* EPI300 cells were incubated in 10 mL LB broth supplemented with 12.5 μg mL^−1^ chloramphenicol at 37°C for 16 h. After incubation, the cells were collected by centrifugation (6,300×*g*, at 4°C for 5 min). Fosmid DNA was purified from the cells with a QIAprep Spin Miniprep Kit (Qiagen, Hilden, Germany). Regarding transposon-based mutagenesis, 2 μL fosmid DNA (100 ng μL^−1^) was mixed with 2 μL EZ-Tn5 Transposon containing a kanamycin-resistant gene (0.002 pmol μL^−1^), 0.5 μL 10× Reaction Buffer, and 0.5 μL EZ-Tn5 Transposase (1U μL^−1^), and then incubated at 37°C for 2 h. The reaction was stopped by adding 1 μL 10× Stop Solution, and incubated at 60°C for 10 min. The mutated fosmid DNA was mixed with *E. coli* EPI300 cells in 0.2 cm-gap Gene Pulser/MicroPulser Cuvettes (Bio-Rad, Hercules, CA, USA), and electroporated at 2.0 kV with a Gene Pulser (Bio-Rad). After electroporation, the cells were suspended in 815 μL of LB broth and allowed to grow at 37°C for 1 h. A portion of the cell suspension (*ca.* 100 μL) was cultured on the LB agar plate supplemented with 12.5 μg mL^−1^ chloramphenicol and 50 μg mL^−1^ kanamycin at 37°C for 1 d. The colonies growing on the plates were replicated on the LB agar plate supplemented with 0.5% tributyrin, 0.1% arabic gum, 12.5 μg mL^−1^ chloramphenicol, and 50 μg mL^−1^ kanamycin. *E. coli* transformants without clear halos represented on LB-tributyrin plate were isolated, and fosmid DNA was purified with a QIAprep Spin Miniprep Kit (Qiagen). The transposon insertion sites were sequenced with the primers KAN-2 FR-1 and RP-1 (Epicentre), a BigDye Terminator v3.1 Cycle Sequencing Kit (Applied Biosystems, Carlsbad, CA, USA), and a 3130*xl* Genetic Analyzer (Applied Biosystems). DNA sequences were analyzed with BLAST and ORF finder programs provided by the National Center for Biotechnology Information (51). The alignment of the translated amino acid sequences and construction of a distance matrix tree on the basis of the neighbor-joining method (50) were performed with the MEGA software (55). According to previous studies on the phylogeny of lipolytic enzymes, the phylogenetic affiliations of the lipolytic enzymes were relatively consistent with the 16S rRNA-based phylogeny (2, 8, 24, 27, 38). Therefore, we compared the phylogenetic diversity of lipolytic bacteria based on the 16S rRNA gene sequences of the culturable isolates and amino acid sequences of the metagenomic clones.

### Nucleotide sequence accession numbers

The nucleotide sequences of the lipolytic enzyme genes and 16S rRNA genes presented in this study have been deposited under DDBJ/EMBL/GenBank accession numbers AB811356 to AB811435.

## Results

### Effect of the oil-fed enrichment strategy on cell counting and soil lipase activity

The moisture content and pH of NS was 7.1 and 60%, respectively. During the enrichment period, moisture content and pH values were maintained at levels of 6.5–7.5 and 55–65%, respectively. The plate counts of lipolytic bacteria in NS and OS accounted for 0.031% and 0.57% of the direct total counts, respectively, indicating that the number of lipolytic bacteria was 18-fold higher in the OS microcosm than in NS microcosm (Table 1). Soil lipolytic activity also increased 4.6-fold after the oil-fed enrichment strategy (Table 1), which demonstrated that lipolytic microorganisms were enriched in the OS microcosm. The bacterial community structures of NS and OS were analyzed by 16S rRNA gene cloning (see Supplemental information). The results obtained showed that the clonal sequences of both microcosms were assigned to eight bacterial phyla (*i.e.*, *Proteobacteria*, *Actinobacteria*, *Acidobacteria*, *Bacteroidetes*, *Verrucomicrobia*, *Planctomycetes*, *Chloroflexi*, and *Nitrospirae*) and hitherto-uncultured bacterial groups (*i.e.*, candidate phyla SPAM and WS3) (Table S1). The Chao1 diversity index based on phylotypes having >97% nucleotide sequence identity was reduced to one-quarter after the oil-fed enrichment.

### Phylogenetic affiliations of culturable lipolytic isolates

We isolated the respective 17 strains from the tributyrin agar plates used for plate counting of lipolytic bacteria in the NS and OS samples, and these were identified by partial 16S rRNA gene sequencing (Table 2). All of the isolates were identified as members of the (sub)phyla *Betaproteobacteria*, *Gammaproteobacteria*, *Firmicutes*, and *Actinobacteria*. The majority of genera to which the isolates from the NS were assigned were associated with *Bacillus* (7 out of 17 strains), *Lysinibacillus* (4 out of 17 strains), and *Pseudomonas* (4 out of 17 strains), and those associated with the genera *Burkholderia* (1 out of 17 strains) and *Rhodococcus* (1 out of 17 strains) were found to be minor constituents. After the enrichment procedure, the lipolytic bacteria assigned to the genera *Burkholderia* (12 out of 17 strains) and *Bacillus* (3 out of 17 strains) dominated in the OS microcosm, except for one strain of *Mitsuaria* (1 out of 17 strains) and one strain of *Curtobacterium* (1 out of 17 strains) (Table 2). These results indicated that the oil-fed enrichment strategy selected particular lipolytic bacteria and excluded other indigenous culturable lipolytic bacteria.

### Screening of lipolytic clones from the metagenomic library

Two fosmid libraries were constructed from the total genomic DNA extracted from NS and OS. The average insert sizes of lipolytic clones obtained from NS and OS were estimated to be 28.1 and 28.6 kbp, amounting to approximately 2.3 and 3.7 Gbp, respectively (Table 1). Among the clones retrieved from NS and OS, 7 and 49 clones showed a clear halo on the LB-tributyrin agar plate. A 4.3-fold increase was observed in the frequency (a ratio of positive lipolytic clones to the total number of clones) after the oil-fed enrichment procedure, suggesting that the oil-fed enrichment strategy was effective at capturing lipolytic metagenomic clones. A total of 56 clones were subjected to subcloning and screening for lipolytic activity in the secondary libraries. All of the 7 clones retrieved from NS were successfully subcloned and sequenced. Of the 49 clones from the OS microcosm library, 39 fosmid clones whose lipolytic activity was confirmed were successfully obtained (Table 3).

### Phylogenetic affiliations of lipolytic enzymes from metagenomic clones

The deduced amino acid sequences of all lipolytic enzymes from the NS and OS metagenomic libraries had high identities with those of the conserved domains of α/β hydrolase, β-lactamase-type hydrolase, and lipase/esterase (Table 3). The majority of lipolytic enzymes from NS was assigned to *Alphaproteobacteria*, *Planctomycetes*, and *Acidobacteria*, and was associated with the families IV, V, and VIII (Fig. S1). On the other hand, those from the OS library were mainly assigned to *Alphaproteobacteria*, *Betaproteobacteria*, *Gammaproteobacteria*, *Deltaproteobacteria*, *Acidobacteria*, *Armatimonadetes*, and *Actinobacteria*, and were associated with the families II, IV, V, and VIII, and the feruloyl esterase group (Fig. S1). The 16S rRNA gene clones classified into these taxa were detected in both the NS and OS communities, except for the members of *Armatimonadetes* (Table S1).

**Fig. 1:**
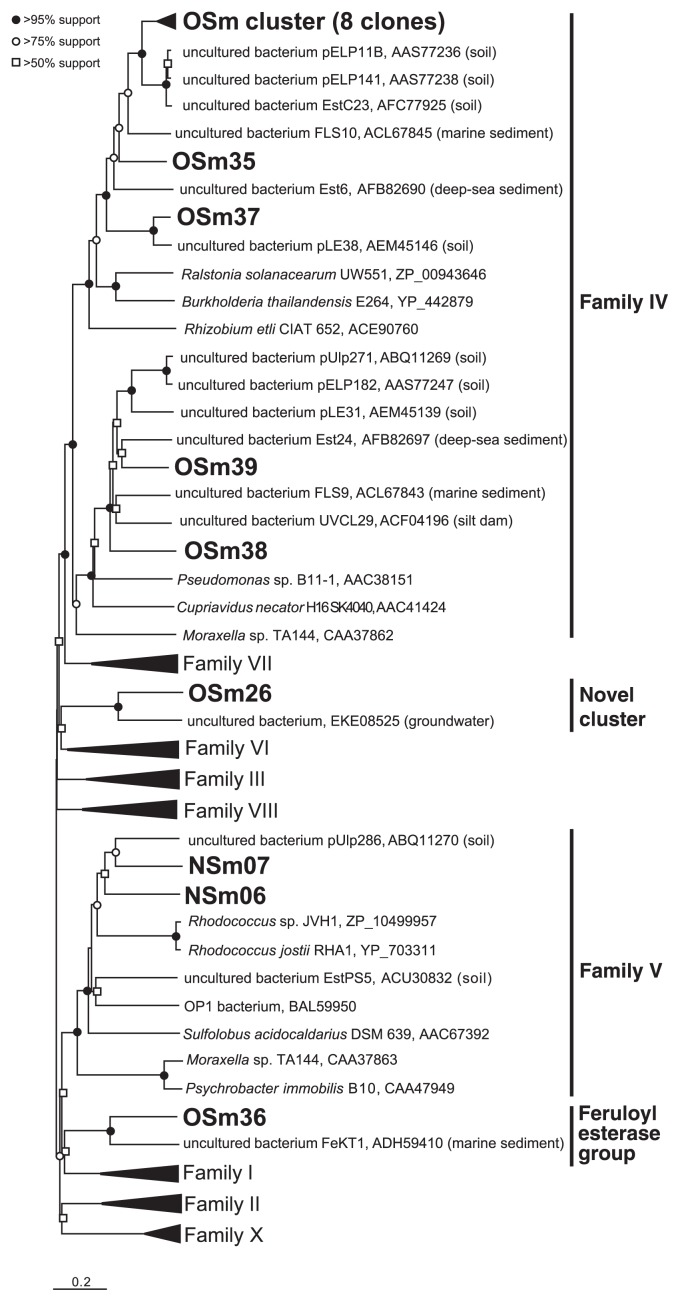
Unrooted distance matrix tree of lipolytic enzymes obtained from non-enriched soil (NS) and oil-enriched soil (OS) metagenomic libraries. The phylogenetic tree was generated by the neighbor-joining method with MEGA 5.0 software. The classification of previously known lipolytic enzymes was based on Arpigny and Jaeger (2). Eight clones from OS (OSm27–34) were assembled into the OSm cluster. The source of lipolytic enzymes related to uncultured bacteria was shown in parenthesis. The topology of the tree was estimated by bootstrap analysis with 1,000 replicates. The bar indicates a 0.2 change per amino acid site.

In addition to the previously known taxa, the functional metagenomic approach enabled us to capture lipolytic enzymes associated with uncultured microorganisms (Table 3). The phylogenetic tree based on amino acid sequences indicated that clones NSm06 and NSm07 obtained from NS were clustered into lipase family V, and had a similar identity to the soil-derived metagenomic clone pUlp286 (53.8% and 50.5% amino acid identities, respectively) (Fig. 1). Within the OS library, a total of 14 clones showed the highest identities to the lipolytic enzymes of uncultured microbes (Table 3), and were divided into three clusters: lipase family IV, the feruloyl esterase group, and a novel cluster (Fig. 1). Twelve clones (OSm27–35 and OSm37–39) were assigned to lipase family IV, and all of these clones had significant identities (61.7%–84.8% amino acid identities) to the lipolytic genes obtained from soil and marine sediment samples. The OSm36 clone had 45.1% amino acid identity with the feruloyl esterase gene of the uncultured bacterium FeKT1 obtained from the marine sediment metagenomic library. In addition, the OSm26clone showed 50.0% amino acid identity to the lipolytic gene found in the groundwater sample.

## Discussion

**Fig. 2:**
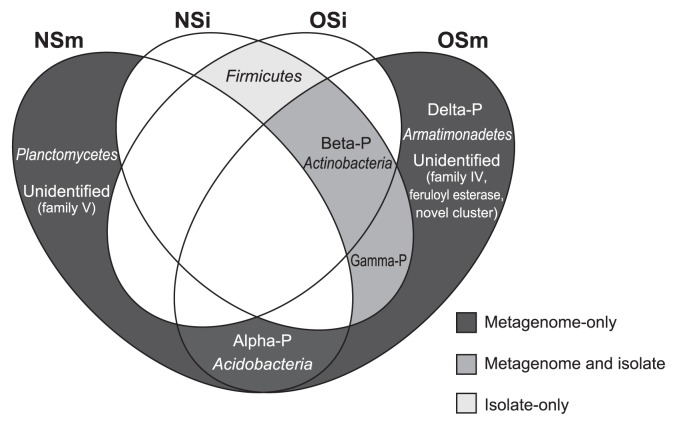
Venn diagram of the phylogeny of lipolytic isolates and genes from non-enriched soil (NS) and oil-enriched soil (OS) metagenomic libraries. Abbreviations: NSi, culturable isolates from NS; OSi, culturable isolates from OS; NSm, metagenomic clones from NS; OSm, metagenomic clones from OS; Alpha-P, *Alphaproteobacteria*; Beta-P, *Betaproteobacteria*; Gamma-P, *Gammaproteobacteria*; Delta-P, *Deltaproteobacteria*.

As reported here, our comparative analyses with two screening approaches (*i.e.*, functional metagenomics and a cultivation-based method) combined with the oil-fed enrichment strategy revealed the entire picture of the phylogenetic diversity of lipolytic bacteria in the forest soil environment, as summarized in a Venn diagram (Fig. 2). One of the most important results was that the functional metagenomic approach detected significant amounts of lipolytic enzymes associated with the phylogenetic groups that were absent in the catalogs of culturable isolates in both NS and OS microcosms; *i.e.*, the (sub)phyla *Alphaproteobacteria*, *Deltaproteobacteria*, *Acidobacteria*, *Armatimonadetes*, and *Planctomycetes*. Within the class *Alphaproteobacteria*, 2 and 12 lipolytic enzymes were identified in NS and OS, respectively. One metagenomic clone associated with *Deltaproteobacteria* and *Armatimonadetes* was retrieved from the OS library only. Within the phylum *Acidobacteria*, 1 and 3 lipolytic enzymes were retrieved from NS and OS, respectively. Two lipolytic enzymes showed the highest identity to those of the phylum *Planctomycetes* in the NS library only. Although several lipolytic enzymes associated with *Alphaproteobacteria*, *Deltaproteobacteria*, *Acidobacteria*, and *Planctomycetes* have been identified in soil metagenomic libraries (6, 12, 16, 35, 38), to the best of our knowledge, there have been no commercially available lipolytic enzymes produced by the culturable strains of these taxa (19, 22). Another important result in our study is that both NS and OS libraries contained a significant proportion of lipolytic enzymes associated with uncultured microorganisms. Based on the phylogenetic analysis, such lipolytic enzymes showed significant identity to the metagenomic clones obtained from soil (28, 36, 38, 46, 53), marine sediment (23, 26), tidal flat sediment (34), and groundwater (62). These results strongly suggest that functional metagenomics combined with the oil-fed enrichment strategy contributed to uncover the phylogenetic diversity of lipolytic bacteria that had been overlooked by the cultivation-based screening technique.

All strains of culturable lipolytic isolates were phylogenetically assigned to three phyla and were further identified as seven genera: *Burkholderia*, *Mitsuaria*, and *Pseudomonas* of the phylum *Proteobacteria; Bacillus* and *Lysinibacillus* of the phylum *Firmicutes*; and *Rhodococcus* and *Curtobacterium* of the phylum *Actinobacteria*. To date, many lipolytic enzymes have been purified from members of the strains of *Bacillus*, *Burkholderia*, and *Pseudomonas* (19, 20, 24, 57). The closest relatives of the isolates classified into the other four genera were also shown to be lipolytic enzyme-producing bacteria (1, 3, 4, 14, 29, 54). Thus, all culturable lipolytic strains were phylogenetically associated with previously characterized lipolytic bacteria. Further studies are needed to compare the biochemical functions (*e.g.*, activity and substrate specificity) of lipolytic enzymes obtained from the culturable isolates and metagenomic libraries.

We did not detect any lipolytic enzymes associated with the phylum *Firmicutes* from either the NS or OS metagenomic library in spite of their presence in the culturable lipolytic bacterial isolates. In addition, the 16S rRNA gene clones classified into this phylum were also undetectable (Table S1). These results indicate that culture conditions needed to isolate the lipolytic bacteria used in this study were suitable for the proliferation of *Firmicutes*, especially the genera *Bacillus* and *Lysinibacillus*. This may also indicate that the metagenomic screening procedure used in this study had potential biases against members of the phylum *Firmicutes*. To overcome this, there is still room for further improvements in the functional metagenomic approach. For example, the host strain may be an important factor in this respect. Previous metagenomic studies reported the use of *Bacillus subtilis* (5), *Pseudomonas putida* (43), *Rhizobium leguminosarum* (61), and *Streptomyces lividans* (11) as host strains as an alternative to *E. coli* to explore novel biocatalysts. Further studies using a functional metagenomic approach with multiple host strains may provide a deeper insight into the phylogenetic diversity of lipolytic microbes in forest soil ecosystems.

## Supplementary Information



## Figures and Tables

**Table 1:** Cell counting, lipase activity, and metagenomic library of non-enriched soil (NS) and oil-enriched soil (OS)

Properties	NS	OS
Total cells (×10^9^ cell g^−1^ [dry wt])^a^	5.3±1.2	2.4±0.1
Culturable lipolytic bacteria (×10^6^ CFU g^−1^ [dry wt])^a^	1.7±0.2	13.8+2.5
The ratio of culturable lipolytic bacteria to the total number of cells (%)	0.031	0.57
Soil lipase activity (U g^−1^ [dry wt])^a^	55.3+7.3	252.8+37.7
Metagenomic libraries		
Total number of clones	80,000	130,000
Average insert size (kb)	28.6	28.1
Estimated total amount of DNA (Gb)	2.29	3.65
Positive lipolytic clones	7	49
The ratio of positive lipolytic clones to the total number of clones (%)	0.00875	0.0377

a Averages and standard deviations were calculated based on 10, 5, and 2 replicates for the total number of cells, culturable lipolytic bacteria, and soil lipase activity, respectively.

**Table 2:** Phylogenetic identification of the lipolytic bacteria isolated from original soil (NS) and oil-enriched soil (OS)

Phylogeny assigned	No. of strains isolated	16S rRNA gene sequence comparison		

		Species as closest relatives	Accession no.	Identity (%)
NS (non-enriched soil)				
Phylum *Firmicutes*				
*Bacillus*	4	*Bacillus simplex* DSM 1321	NR_042136	99.6–100
	2	*Bacillus megaterium* NBRC 13498	AB680420	99.7–100
	1	*Bacillus thuringiensis* serovar *kurstaki* HD73	CP004069	99.9
*Lysinibacillus*	4	*Lysinibacillus sphaericus* 205y	AF435435	99.7–100
Phylum *Proteobacteria*				
Class *Betaproteobacteria*				
*Burkholderia*	1	*Burkholderia phytofirmans* PsJN	CP001052	99.1
Class *Gammaproteobacteria*				
*Pseudomonas*	1	*Pseudomonas arsenicoxydans* MaBP1	JQ317810	99.3
	1	*Pseudomonas putida* ATCC 17472	AF094738	99.6
	1	*Pseudomonas putida* NBRC 102093	AB681704	98.7
	1	*Pseudomonas vancouverensis* DhA-51	AJ011507	99.7
Phylum *Actinobacteria*				
*Rhodococcus*	1	*Rhodococcus erythropolis* CR-53	AJ786263	99.4

OS (Oil-enriched soil)				
Phylum *Firmicutes*				
*Bacillus*	2	*Bacillus thuringiensis* serovar *kurstaki* HD73	CP004069	99.5–99.8
	1	*Bacillus simplex* DSM 1321	NR_042136	100
Phylum *Proteobacteria*				
Class *Betaproteobacteria*				
*Burkholderia*	10	*Burkholderia caledonica* LMG 19076	HQ849076	97.9–99.8
	1	*Burkholderia hospita* LMG 20598	HQ849087	99.1
	1	*Burkholderia glathei* N15	NR_037065	97.9
*Mitsuaria*	1	*Mitsuaria chitosanitabida* NBRC 102408	AB681764	98.8
Phylum *Actinobacteria*				
*Curtobacterium*	1	*Curtobacterium herbarum* DSM 14013	AM410692	96.0

**Table 3:** Phylogenetic identification of the lipolytic metagenomic clones from original soil (NS) and oil-enriched soil (OS)

Phylogeny assigned	Clone no.	Closest relatives	Accession no.	Product	Identity (%)	Family/Group^a^
NS (non-enriched soil)						
*Alphaproteobacteria*	NSm01	*Parvibaculum lavamentivorans* DS-1	YP_001411752	α/β hydrolase	54.3	IV
	NSm02	*Methylobacterium nodulans* ORS 2060	YP_002501216	β-lactamase	47.2	VIII
*Planctomycetes*	NSm03	*Planctomyces maris* DSM 8797	ZP_01853484	α/β hydrolase	52.4	IV
	NSm04	*Pirellula staleyi* DSM 6068	YP_003372868	α/β hydrolase	39.9	V
*Acidobacteria*	NSm05	“*Candidatus* Solibacter usitatus” Ellin6076	YP_825272	α/β hydrolase	77.8	V
Unidentified groups	NSm06	uncultured bacterium clone pUlp286	ABQ11270	Lipase/esterase	53.8	V
	NSm07	uncultured bacterium clone pUlp286	ABQ11270	Lipase/esterase	50.5	V

OS (oil-enriched soil)						
*Alphaproteobacteria*	OSm01	*Tistrella mobilis* KA081020-065	YP_006374406	β-lactamase	55.0	VIII
	OSm02	*Caulobacter* sp. K31	YP_001682441	β-lactamase	92.2	VIII
	OSm03	*Methylobacterium radiotolerans* JCM 2831	YP_001752969	α/β hydrolase	37.1	IV
	OSm04	*Methylobacterium nodulans* ORS 2060	YP_002498598	β-lactamase	58.7	FE
	OSm05	*Parvibaculum lavamentivorans* DS-1	YP_001411752	α/β hydrolase	58.5	IV
	OSm06	*Hyphomonas neptunium* ATCC 15444	YP_759849	Putative esterase	69.8	VIII
	OSm07	*Bradyrhizobium diazoefficiens* USDA 110	NP_770666	β-lactamase	86.2	VIII
	OSm08	*Hyphomonas neptunium* ATCC 15444	YP_759753	Putative esterase	58.6	VIII
	OSm09	*Sphingomonas echinoides* ATCC 14820	ZP_10338216	β-lactamase	70.8	VIII
	OSm10	*Mesorhizobium opportunistum* WSM2075	ZP_05809008	α/β hydrolase	53.5	V
	OSm11	*Rhizobium tropici* CIAT 899	YP_007335702	α/β hydrolase	50.0	IV
	OSm15	*Rhodopseudomonas palustris* HaA2	YP_486641	Esterase/lipase/thioesterase	59.0	IV
*Betaproteobacteria*	OSm12	*Burkholderia gladioli* ATCC 10248	AAF59826	Esterase estB	57.7	VIII
	OSm13	*Burkholderia gladioli* ATCC 10248	AAF59826	Esterase estB	58.1	VIII
	OSm14	*Burkholderia thailandensis* MSMB43	ZP_02464527	Putative lipoprotein	35.4	FE
	OSm16	*Cupriavidus basilensis* OR16	ZP_09622859	β-lactamase	81.4	VIII
*Gammaproteobacteria*	OSm17	*Pseudomonas synxantha* BG33R	ZP_10142349	Esterase estA	100	II
	OSm18	*Pseudomonas synxantha* BG33R	ZP_10142349	Esterase estA	97.3	II
	OSm19	*Xanthomonas albilineans* GPE PC73	YP_003374881	Carboxylesterase bioH	57.7	V
*Deltaproteobacteria*	OSm20	*Desulfococcus oleovorans* Hxd3	YP_001530546	β-lactamase	51.1	VIII
*Actinobacteria*	OSm21	*Streptomyces venezuelae* ATCC 10712	YP_006876070	Esterase	55.3	VIII
*Armatimonadetes*	OSm22	*Chthonomonas calidirosea* T49	YP_008088291	Hydrolase	26.5	V
*Acidobacteria*	OSm23	“*Candidatus* Koribacter versatilis” Ellin345	YP_589716	β-lactamase	43.8	VIII
	OSm24	“*Candidatus* Koribacter versatilis” Ellin345	YP_589716	β-lactamase	51.0	VIII
	OSm25	“*Candidatus* Solibacter usitatus” Ellin6076	YP_825272	α/β hydrolase	80.1	V
Unidentified groups	OSm26	uncultured bacterium ACD_17C00118G0001	EKE08525	Hypothetical lipase	50.0	NC
	OSm27	uncultured bacterium pELP141	AAS77238	Lipase/esterase	77.2	IV
	OSm28	uncultured organism EstC23	AFC77925	Lipase/esterase	74.8	IV
	OSm29	uncultured bacterium pELP141	AAS77238	Lipase/esterase	64.3	IV
	OSm30	uncultured bacterium pELP141	AAS77238	Lipase/esterase	77.8	IV
	OSm31	uncultured bacterium pELP11B	AAS77236	Lipase/esterase	76.7	IV
	OSm32	uncultured bacterium pELP141	AAS77238	Lipase/esterase	79.5	IV
	OSm33	uncultured organism EstC23	AFC77925	Lipase/esterase	77.1	IV
	OSm34	uncultured organism EstC23	AFC77925	Lipase/esterase	75.9	IV
	OSm35	uncultured bacterium FLS10	ACL67845	Lipolytic enzyme	67.4	IV
	OSm36	uncultured bacterium FeKT1	ADH59410	Feruloyl esterase	45.1	FE
	OSm37	uncultured bacterium pLE38	AEM45146	Lipase/esterase	84.8	IV
	OSm38	uncultured bacterium UVCL29	ACF04196	Lipase/esterase	62.7	IV
	OSm39	uncultured bacterium Est24	AFB82697	Lipase/esterase	61.7	IV

a The definition of the families (or groups) of lipolytic enzymes were according to previous studies (2, 8, 23, 35, 39): II, IV, V, and VIII, number of the family; FE, feruloyl esterase group; NC, novel cluster (shown in Fig. 1 and Fig. S1).
