# Supplementary material for: The Combination of Functional Metagenomics and an Oil-Fed Enrichment Strategy Revealed the Phylogenetic Diversity of Lipolytic Bacteria Overlooked by the Cultivation-Based Method
Source: Microbes Environ. 2014 May 23;29(2):154–61. doi: 10.1264/jsme2.ME14002 (PMC4103521; doi:10.1264/jsme2.ME14002)
Supplement: Supplementary file 1 [file 29_154_s1.pdf]

Supplemental information

Materials and Methods

Bacterial 16S rRNA gene clone library

DNA extraction from NS and OS samples were performed by using FastDNA SPIN Kit for Soil (MP Biomedicals) according to manufacturer's instruction. The bacterial 16S rRNA gene was amplified with the primers EUB338mix (2) and Univ1492r (3) according to a previously described method (5). PCR amplicons were purified with a QIAquick PCR Purification Kit (Qiagen, Valencia, CA) and subcloned into a pT7Blue-2 T-vector (Novagen, Madison, WI) by using a DNA Ligation kit (TaKaRa Bio, Otsu, Japan) according to the manufacturer's instructions. More than 100 clones were randomly retrieved from each library. Cloned genes were partially sequenced (ca. 400-500 bp) with a BigDye Terminator v3.1 Cycle Sequencing Kit (Applied Biosystems, Foster City, CA) and a 3130*xl* Genetic Analyzer (Applied Biosystems). Partial-length sequences were aligned and inserted into the tree using the parsimony tool of the ARB program (4). Phylogenetic assignment of each phylotype was determined by BLAST program provided by the National Center for Biotechnology Information (6). The phylotype was defined as a group of cloned sequences with >97.0% nucleotide sequence identity for the bacterial 16S rRNA gene. The Chao1 nonparametric species richness estimator was used for the evaluation of α-diversity of NS and OS microcosms (1).

Figure legends

Fig. S1. Unrooted distance matrix tree of lipolytic enzymes obtained from non-enriched soil (NS) and oil-enriched soil (OS) metagenomic libraries. The phylogenetic tree was generated by maximum likelihood with MEGA 5.0 software. The classification of previously known lipolytic enzymes was based on previous studies (references nos. in the main text: 2, 8, 23, 35, 39). Eight clones from OS (OSm27–34) were assembled into OSm cluster. Bar indicates 0.5 change per amino acid site.

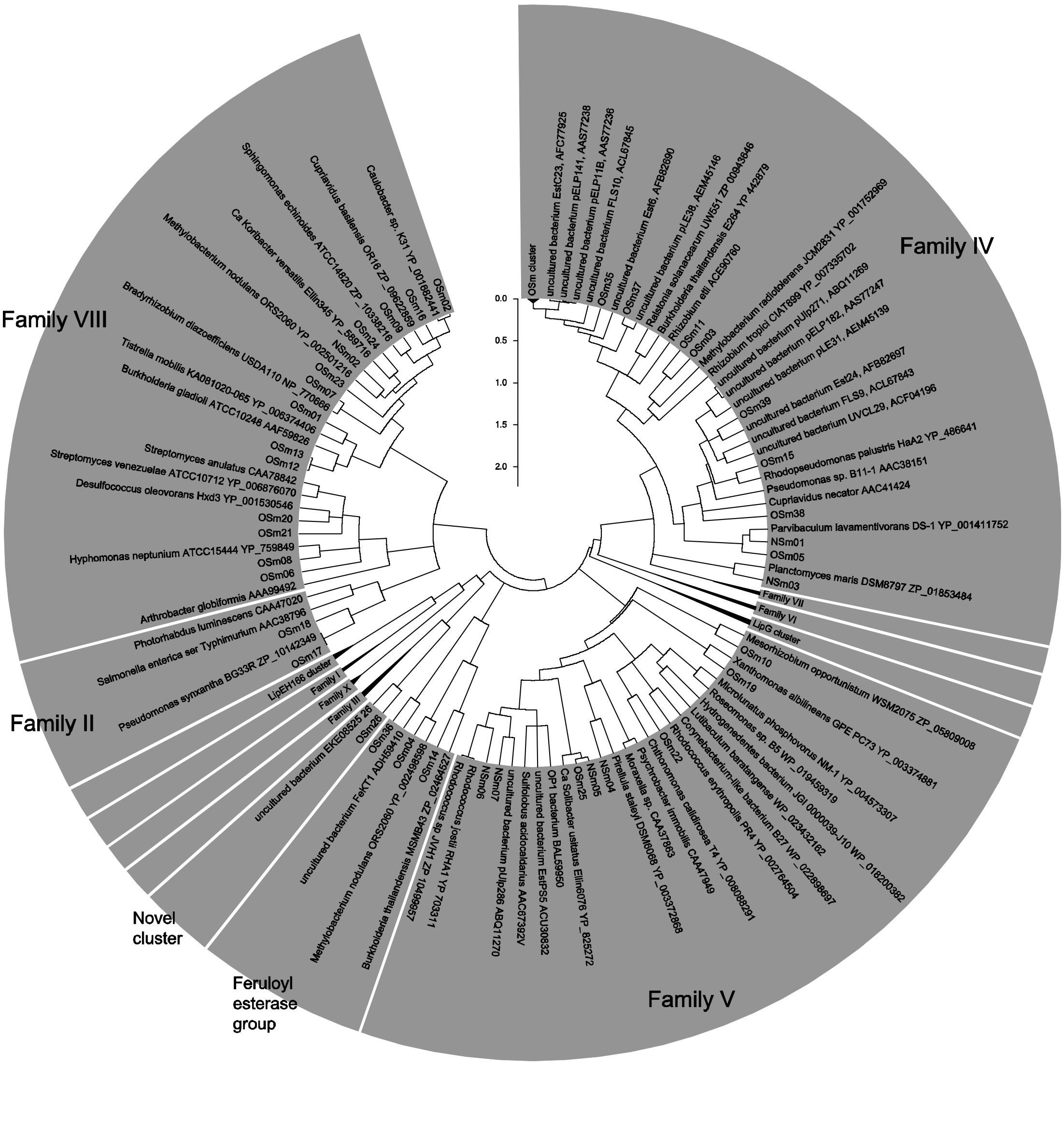

Table S1. Bacterial clone libraries of NS and OS microcosms

	NS	OS
Clone numbers	144	125
OTU (>97% homology)	113	85
Chao1 estimator	799	201
Phylum and class assigned[a]	Relative abundance (%)	Relative abundance (%)
Proteobacteria		
Alphaproteobacteria	19.4	26.4
Betaproteobacteria	7.6	11.2
Gammaproteobacteria	14.6	12
Deltaproteobacteria	9.0	4.0
Actinobacteria	16.0	12.8
Acidobacteria	11.1	12.8
Bacteroidetes	7.6	6.4
Verrucomicrobia	6.3	2.4
Planctomycetes	2.1	3.2
Chloroflexi	3.5	0.8
Nitrospirae	0.7	0
Clone clusters		
SPAM	0.7	4.8
WS3	1.4	1.6
Unidentified cluster	0	1.6

[a] Numbers indicated relative abundances (%) for each phylogenetic group.

Supplemental References

1. Chao, A. 1987. Estimating the population size for capture-recapture data with unequal catchability. Biometrics. 43:783-791.
2. Daims, H., A. Bruhl, R. Amann, K.H. Schleifer, and M. Wagner. 1999. The domain-specific probe EUB338 is insufficient for the detection of all Bacteria: Development and evaluation of a more comprehensive probe set. Systematic and Applied Microbiology. 22:434-444.
3. Lane, D.J. 1991. 16S/23S rRNA sequencing, p. 115-175. *In* E. Stackebrandt, and M. Goodfellow (ed.), Nucleic acid techniques in bacterial systematics. John Wiley & Sons, Chichester, United Kingdom.
4. Ludwig, W., O. Strunk, R. Westram*, et al.* 2004. ARB: a software environment for sequence data. Nucleic Acids Res. 32:1363-1371.
5. Narihiro, T., T. Terada, K. Kikuchi*, et al.* 2009. Comparative analysis of bacterial and archaeal communities in methanogenic sludge granules from upflow anaerobic sludge blanket reactors treating various food-processing, high-strength organic wastewaters. Microbes Environ. 24:88-96.
6. Sayers, E.W., T. Barrett, D.A. Benson*, et al.* 2011. Database resources of the National Center for Biotechnology Information. Nucleic Acids Res. 39:D38-D51.
